# Annual acknowledgement of manuscript reviewers

**DOI:** 10.1186/s13690-015-0063-2

**Published:** 2015-03-31

**Authors:** Herman Van Oyen, Olivier Bruyère

**Affiliations:** Public Health and Surveillance, Scientific Institute of Public Health, Brussels, Belgium; Department of Public Health, Epidemiology and Health Economics, University of Liege, Liege, Belgium

## Abstract

The editors of *Archives of Public Health* would like to thank all our reviewers who have contributed to the journal in Volume 72 (2014).

**Goutam Adak**

United Kingdom

**Willem Aelvoet**

Belgium

**Allison Agwu**

United States of America

**S. Faisal Ahmed**

United Kingdom

**Mehrnoosh Akhtari Zavare**

Malaysia

**Mamusha Aman**

Ethiopia

**Julie Armin**

United States of America

**Forest Arnold**

United States of America

**Wouter Arrazola de Onate**

Belgium

**Colin Baker**

United Kingdom

**Ivan Bautmans**

Belgium

**Chalotte Beaudart**

Belgium

**Sarah Bel**

Belgium

**Rhonda BeLue**

United States of America

**Jeroen Bergmann**

United Kingdom

**Paloma Bonato**

Brazil

**Nathalie Bossuyt**

Belgium

**Maria Luisa Brandi**

Italy

**Hannah Brinsden**

United Kingdom

**Henrik Bronnum-Hansen**

Denmark

**Olivier Bruyère**

Belgium

**Frank Buntinx**

Belgium

**Caroline Burgess**

United Kingdom

**Vincent Busch**

Netherlands

**Guido Calleri**

Italy

**Adrian Cameron**

Australia

**Martin Caraher**

United Kingdom

**Bin Chen**

China

**Natalie Colabianchi**

United States Minor Outlying Islands

**Elizabeth Conrey**

United States of America

**Anna Cooper**

United Kingdom

**Cyrus Cooper**

United Kingdom

**Joana Costa**

Portugal

**Michelle Crino**

Australia

**Claudia Crowell**

United States of America

**Deborah Cuignet**

Belgium

**Dinny de Bakker**

Netherlands

**Willem De Keyzer**

Belgium

**Jessika Deblonde**

Belgium

**Anja Declercq**

Belgium

**Stefaan Demarest**

Belgium

**Henriëtte Dijkshoorn**

Netherlands

**Jean Donadieu**

France

**Nülüfer Erbil**

Turkey

**Fatma Ersin**

Turkey

**Knut Fylkesnes**

Norway

**Isabelle Godin**

Belgium

**Tulika Goswami Mahanta**

India

**Nathan Green**

United Kingdom

**Nicholas Harvey**

United Kingdom

**Mickael Hiligsmann**

Netherlands

**Perrine Humblet**

Belgium

**Rafaele Huntjens**

Netherlands

**Thomas Janssens**

Belgium

**Ximiao Jiang**

United States of America

**Nkomba Kayeyi**

Norway

**Solen Kernéis**

France

**Tadek Krzywania**

Belgium

**Fumiaki Kura**

Japan

**Oliver Kuss**

Germany

**Hannah Lambie-Mumford**

United Kingdom

**Arnaud Le Menach**

United States of America

**Vasiliki Leventakou**

Greece

**Mary Litchford**

United States of America

**Joe Lugalla**

United States of America

**Dave MacLeod**

United Kingdom

**Sandra Mandic**

New Zealand

**Joseph Manzambi Kuwekita**

Belgium

**Airin Denise Martinez**

United States of America

**Herman Meulemans**

Belgium

**Etsuko Miyagi**

Japan

**Michal Molcho**

Ireland

**Theodora Mouratidou**

Italy

**Alexandre Mouton**

Belgium

**Cory Neudorf**

Canada

**Christiana Noestlinger**

Belgium

**Aleck Ostry**

Canada

**Edwin Pelfrene**

Belgium

**Eero Pukkala**

Finland

**Virginia Quick**

United States of America

**Magdalena Raban**

Australia

**Jean-Yves Reginster**

Belgium

**Kieran Reid**

United States of America

**Francoise Renard**

Belgium

**Francoise Renard**

Belgium

**Giampaolo Ricci**

Italy

**Ward Schrooten**

Belgium

**Ian Sheerin**

New Zealand

**Lee So Lun**

Hong Kong

**Catherine Stewart**

United Kingdom

**Véronique Tellier**

Belgium

**Jürgen Thelen**

Germany

**Viviane Van Casteren**

Belgium

**Aurelie Van Hoye**

France

**Carine Van Malderen**

Belgium

**Hadewijch Vandenheede**

Belgium

**Katrien Vanthomme**

Belgium

**Pierre Verger**

France

**Eric Vermetten**

Netherlands

**Marieke Verschuuren**

Netherlands

**Jean-Louis Vincent**

Belgium

**Jo Waller**

United Kingdom

**Wilma Waterlander**

New Zealand

**Allison Webel**

United States of America

**Tebikew Yeneabat**

Ethiopia

**Renata Yokota**

Belgium

**Joris Yzermans**

Netherlands

**Joris Yzermans**

Netherlands

**Lihui Zhang**

Canada

